# Comparative genomic analysis of food-animal-derived and human-derived *Clostridium perfringens* isolates from markets in Shandong, China

**DOI:** 10.3389/fmicb.2025.1543511

**Published:** 2025-04-01

**Authors:** Xinyang Zhu, Yucui Huang, Yuxia Shi, Xiaojie Gao, Duanduan Chen, Cheng Liu, Shengliang Cao, Xijuan Xue, Yubao Li

**Affiliations:** ^1^College of Agriculture and Biology, Liaocheng University, Liaocheng, Shandong, China; ^2^Shandong Sinder Technology Co., Ltd., Weifang, Shandong, China; ^3^School of Pharmaceutical Sciences and Food Engineering, Liaocheng University, Liaocheng, Shandong, China

**Keywords:** *Clostridium perfringens*, whole genome sequencing, multidrug-resistant bacteria, toxinotypes, pangenome analysis

## Abstract

*Clostridium perfringens* (*C. perfringens*) is a foodborne pathogen that poses a significant threat to both animal husbandry and public health. In this study, 27 *C. perfringens* strains were isolated from animal samples and animal-derived food products. Antibiotics resistances among the isolates were phenotypically and genotypically analyzed and Whole genome sequencing (WGS). In combination with the genomes of 141 human-derived *C. perfringens* strains from public databases, this study conducted comprehensive analyses of antibiotic resistance genes, virulence genes, multilocus sequence typing (MLST), prophage detection, and pan-genome analysis for a total of 168 strains of *C. perfringens*. Antibiotics resistances among the isolates were phenotypically and genotypically analyzed and found 24 of them (88.9%, 24/27) were identified as multidrug-resistant (MDR). WGS analysis revealed that 13 strains belonged to known sequence types (ST), and the remaining strains represented 10 new STs. By analysis in combination with data of 141 *C. perfringens* isolates from the database, it was implied that ST221, ST72 and ST370 were present in both animal-derived and human-derived *C. perfringens*. It is worth noting that 108 out of 168 strains of *C. perfringens* (64.3%, 108/168) were found to carry prophages, which were found more prevalent in human-derived *C. perfringens* isolates. Pan-genome and phylogenetic analysis of 168 *C. perfringens* strains indicated that *C. perfringens* possesses an open pan-genome with genetic diversity. This study provides genomic insights into *C. perfringens* from food animals and humans, shedding light on the importance for monitoring the *C. perfringens* in livestock in China for better public health.

## 1 Introduction

*Clostridium perfringens (C. perfringens)* is an anaerobic pathogen that belongs to Gram-positive bacteria. It is generally found in environments and animals and is regarded as one of the main determinants of gastrointestinal disorders in both humans and livestock (Scallan et al., 2011). This bacterium is primarily responsible for necrotic enteritis and enterotoxemia in animals especially in poultry, while in humans, it is often associated with food poisoning and gas gangrene (Guillaume et al., 2017). *C. perfringens* is known to produce over 20 different types of exotoxins which are considered to be their principal virulence factors (Rood et al., 2018), wherein the key toxins including α, β, ε, iota, cpe, and NetB toxin are of primary concerns (Navarro et al., 2018). *C. perfringens* can be classified into seven types (A - G) based on the carriage of these key toxins.

According to the Centers for Disease Control and Prevention (CDC), *C. perfringens* is ranked among the five most common food-borne pathogens in the United States (Ha et al., 2019). Outbreaks of *C. perfringens* have also been reported in meat retail establishments (Wittry et al., 2022). For instance, there were several reports of human food poisoning caused by *C. perfringens* in the United States in 1983 (Shandera et al., 1983). Additionally, reports indicated the presence of *C. perfringens* in from retail beef (Jiang et al., 2021), chicken (Xu et al., 2021) and duck (Liu et al., 2020) meat in China. In recent years, there have also been patients infected by *C. perfringens* that reported in Guangzhou (Huang et al., 2023), Hangzhou (Yan et al., 2024), and central China (Wang et al., 2021). Studies have demonstrated that *C. perfringens* are able to colonize in the intestines of newborns within 2 days post birth (Huang et al., 2023), and can cause severe neonatal necrotic enteritis (Li et al., 2022). Notably, the prevalence of drug resistance in *C. perfringens* has been increasing, particularly the animal-derived *C. perfringens* have been frequently found to be multidrug-resistant (Derongs et al., 2020; Liu et al., 2020). Considering their capability to transmit to anthropogenic community via food chain, these multidrug-resistant strains are thought to pose a substantial threat to human wellbeing and public health (Khandia et al., 2021).

The widespread application of high-throughput sequencing technology has greatly promoted the in-depth understanding toward *C. perfringens*. For example, a research in 2002 revealed that the relatively low GC contents in the genome of *C. perfringens* and confirmed that the presence of typical anaerobic fermentation enzymes responsible for gas production on its genome (Shimizu et al., 2002). In addition, further studies elucidating the virulence genes of *C. perfringens* has improved our understanding to their mechanisms associated with pathogenicity (Fourie et al., 2020; Mehdizadeh Gohari et al., 2021). And based on whole-genome analysis, researchers have established a new multi-locus sequence typing (MLST) method which provides a new perspective for accurately profiling the isolates (Abdel-Glil et al., 2021b). MLST is a well-established and widely used method for bacterial typing and phylogenetic analysis. It is based on the polymorphism of conserved housekeeping genes, which are essential for bacterial survival and are less likely to be affected by horizontal gene transfer (Jolley et al., 2018). This characteristic ensures MLST’s reliability and portability for cross-laboratory strain comparisons. However, MLST exhibits limitations in discriminatory power, particularly when differentiating closely related strains (Hepner et al., 2025). In contrast, genome-wide analyses such as core-genome methods provide greater resolution and accuracy in inferring evolutionary relationships by utilizing single nucleotide variations (SNPs). This approach captures finer genetic distinctions between strains. Compared to MLST, core-genome techniques are more effective at distinguishing strains that share the same sequence type (ST), offering enhanced sensitivity in tracing pathogen transmission routes and conducting epidemiological investigations (Yan et al., 2023). Some studies have pointed out that only 12.6% of the *C. perfringens* genome is assigned as the core genome, which indicated that *C. perfringens* possesses a pan-genome diversity (Kiu et al., 2017). Recent genomic and comparative studies on *C. perfringens* highlight its dynamic evolution and virulence mechanisms (Abdel-Glil et al., 2021a). Advances in pan-genome analyses reveal extensive toxin gene diversity linked to strain pathogenicity, driven by plasmid-mediated horizontal gene transfer (Gulliver et al., 2023). Comparative genomics underscores host-specific adaptations, such as poultry-associated necrotic enteritis strains harboring unique virulence loci (Meniaï et al., 2021). These findings unveil the valuable insights into the diversity and heterogeneity of *C. perfringens*.

In this study, we investigated the genome sequences of 27 *C. perfringens* strains isolated from Shandong, China, and comprehensively analyzed the genetic determinants of antibiotic resistances or virulences. Subsequently, the genome data of 141 human-derived *C. perfringens* were obtained from NCBI which have been subjected to a comparative genomic analysis.

## 2 Materials and methods

### 2.1 Isolation of *C. perfringens* strains

Between 2021 and 2022, *C. perfringens* isolates were recovered from retail meat products in three cities of Shandong Province, China (Liaocheng, Weifang, and Linyi). Among 88 analyzed samples comprising 36 pork and 52 poultry, *C. perfringens* was detected in 19.4% (7/36) of pork and 38.5% (20/52) of poultry samples, totaling 27 isolates. The samples were carefully homogenized, then inoculated onto solid tryptone-sulfite-cycloserine agar (TSC, Qingdao Hope Bio-Technology Co., Ltd). The plates were incubated under anaerobic conditions at 37°C for 24 h, after which single colonies exhibiting the morphological characteristics of *C. perfringens* were selected and re-inoculated onto additional TSC plates. The selected single colonies were then transferred to brain heart infusion (BHI, Qingdao Hope Bio-Technology Co., Ltd) and cultured under anaerobic conditions at 37°C for 12 h. To profile the toxins, DNA were extracted the samples and applied for the polymerase chain reaction (PCR) using primers listed in Supplementary Table 1 (Miyamoto et al., 2012). Finally, the identified strains were kept and stored at −80°C. Additionally, we obtained *C. perfringens* genomes of Chinese origin deposited in the NCBI database (*n* = 141) (Supplementary Table 2).

### 2.2 DNA extraction, sequencing, and annotation

Genomic DNA from *C. perfringens* was extracted using the FastPure Bacteria DNA Isolation Mini Kit-Box 2 (Nanjing Vazyme Biotechnology Co., Ltd.). Whole-genome sequencing was carried out by Novogene Technology Co., Ltd. (Beijing, China) using Illumina Novaseq-PE150 platform. All data were processed using fastp v0.23.4 to trim adapter and low-quality reads (Chen et al., 2018), and FastQC v0.12.1 (Wingett and Andrews, 2018). The clean reads were then assembled using Shovill v1.1.0^1^ to construct genome assembly. The quality of genome assemblies was determined using Quast v5.2.0 (quast.py contigs.fasta) (Gurevich et al., 2013), and genome completeness/contamination was evaluated using CheckM v1.2.0 (Parks et al., 2015). A high-quality genome is defined as one exceeding 90% completeness with contamination levels below 5% (Parks et al., 2020). Genome annotation was performed using the NCBI Prokaryotic Genome Annotation Pipeline (PGAP v6.4) (Tatusova et al., 2016).

### 2.3 Antimicrobial susceptibility testing

All antibacterial tablets used in this study were obtained from Hangzhou Microbial Reagent Co., Ltd. (Hangzhou, China). The susceptibility of *C. perfringens* to nine antibiotics from six different classes was assessed: lincomycin (2 μg), cefotaxime (30 μg), neomycin (30 μg), florfenicol (30 μg), amoxicillin (20 μg), tetracycline (30 μg), norfloxacin (10 μg), bacitracin (0.04 IU), and spectinomycin (100 μg). *C. perfringens* ATCC 13124 was used as a quality control strain. The susceptibility of these strains was categorized as resistant, moderately susceptible, or susceptible according to the Clinical and Laboratory Standards Institute (CLSI) criteria^2^ (Humphries et al., 2021). Multidrug-resistant bacteria (MDR) was defined as acquired non-susceptibility to at least one agent in three or more antimicrobial categories (Magiorakos et al., 2012).

### 2.4 Virulence genes, ARGs and prophage prediction

The comparison of the *C. perfringens* genome was conducted using the Pathogen Virulence Factors Database (VFDB) database^3^ to identify the virulence factors (Bo et al., 2019). To predict the antibiotic resistance genes (ARGs), the Resistance Gene Identifier (RGI v6.0.2) tool was employed in combination with the Comprehensive Antibiotic Resistance Database (CARD v4.0.0)^4^ (Alcock et al., 2023). To minimize the inclusion of mis-assembled or mis-annotated antibiotic resistance genes (ARGs), we excluded protein-coding genes that met either of the following criteria: less than 70% amino acid sequence identity to the corresponding CARD reference ARG, or sequence length falling outside the 90%–130% range relative to the reference sequence (Wang and Dagan, 2024; Supplementary Table 3). Additionally, we utilized the online toolkit PHASTEST^5^ (David et al., 2016), to predict the loci of prophages within the *C. perfringens* and genome and to analyze the genomic characteristics of these prophages. PHASTEST scores are determined based on the number of coding sequences (CDS) present in the DNA sequence as well as the presence or absence of phage-related genes. Predicted prophage sequences were categorized into three categories: complete, putative, and incomplete. Heatmap visualization and similarity-based clustering analysis of the data were performed using TBtools-II (Chen et al., 2023).

### 2.5 MLST and SNP analysis

The genomes of 27 *C. perfringens* strains were subjected to the PubMLST *C. perfringens* database (Jolley et al., 2018)^6^ and compared with the alleles and sequence types (ST) deposited in the database for multi-locus sequence typing (MLST) analysis. The online website CSI Phylogeny 1.4^7^ (Kaas et al., 2014) was used to determine the SNPs and constructs core genome phylogeny through concatenated high-quality SNP alignments. The visualization was achieved using iTOL v6^8^ (Letunic and Bork, 2021).

### 2.6 Pan-genome analysis of *C. perfringens*

To study the phylogenetic relationships among *C. perfringens*, the GFF3 files of 168 isolates we generated using the script *bp_genbank2gff3.pl* were used as Roary (v3.13.0) (Page et al., 2015). To identify potential false-positive clusters, examine the Min group, Max group, and Avg group columns in the gene_presence_absence.csv file generated by Roary for discrepancies in gene length. If significant discrepancies are detected, extract the target cluster sequences from pan_genome.fasta, perform multiple sequence alignment using MAFFT (v.7.526) (Katoh et al., 2019) to assess consistency, and finally remove confirmed false-positive clusters from the gene_presence_absence.csv file. The input for extracting and aligning core genes (core_gene_alignment.aln) was processed with an 95% threshold to differentiate core genomes from non-essential and strain-specific genomes, for pan-genome and core gene analysis of all strains. A phylogenetic tree of *C. perfringens* was then constructed based on the core genome sequence using MEGA 11 software (Tamura et al., 2021), subsequently visualized by iTOL v6 (Letunic and Bork, 2021).

### 2.7 Statistical analysis

All statistical analyzes were performed using the GraphPad Prism 9.0 (GraphPad Software Inc., San Diego, CA, USA) and SPSS version 19.0 (IBM Corporation, USA). The concordance between ARGs and antimicrobial resistance phenotypes was evaluated using the kappa coefficient as documented previously (Landis and Koch, 1977).

## 3 Results

### 3.1 Genomic analysis

Among the 24 of the total *C. perfringens* strains were isolated from poultry meat and intestines, while the other 3 were identified in the pork samples. Of these, 26 isolates were classified as type A, with only 1 strain was classified as type G (Supplementary Table 4). The genome sizes of the *C. perfringens* strain from 3.1 to 3.5 Mbp, with GC contents between 28 and 28.5%. The completeness of the genomes ranged from 92.6 to 98.9%, while the contamination levels were observed to be between 2.4 and 4.8%. Additionally, the N50 values varied significantly, ranging from 78,831 to 2,071,785 bp. The sequencing data have been deposited in NCBI, which was accessible from the accession numbers listed in Supplementary Table 5.

### 3.2 Profiling of phenotypic and genotypic antibiotic resistances

**FIGURE 1:**
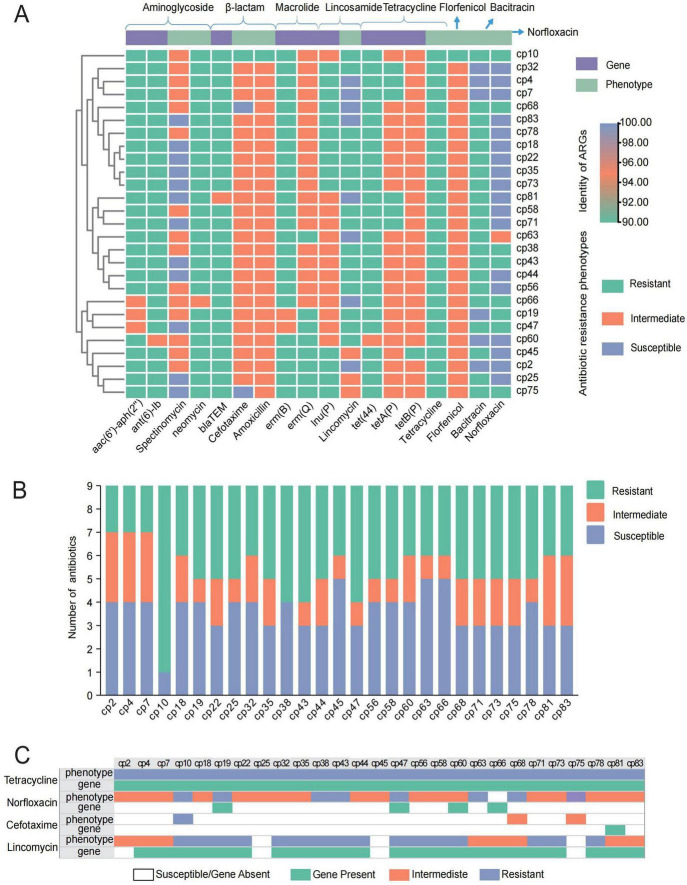
Results of *C. perfringens* resistance analysis. **(A)** Heatmap: antimicrobial resistance phenotypes and predicted ARGs; Dendrogram: clustering rows with similar data. **(B)** Antibiotic resistance of each *C. perfringens* strain; **(C)** comparison of drug-resistant phenotypes and ARGs correlations in 27 strains of *C. perfringens*.

A total of nine resistance genes were identified in sequencing data of 27 *C. perfringens* isolates (Figure 1A). All strains were found to harbor one to five resistance genes, wherein the tetracycline resistance genes *tetA* (P) and *tetB* (P) were most prevalent (74.7 and 96.2%). Approximately 48.1% of the strains carried the *lnu* (P) resistance gene, which is responsible for the inactivation of lincosamide antibiotics. Additionally, there are two types of genes that mediated the resistances to macrolides. Specifically, 70.4% of the isolates were found with *erm* (Q) gene, while 7.4% of them contained the *erm* (B) gene. All 27 strains of *C. perfringens* isolates exhibited high phenotypic resistances to tetracycline, neomycin, and bacitracin, yet demonstrating sensitivities to cefotaxime, amoxicillin, and florfenicol. We also found that 24 isolates were classified as multidrug-resistant (MDR) by mediating resistance to several antibiotics of different classes (Figure 1B). Of note, the tight association between resistance phenotypes and genotypes was only observed in tetracycline class, with a kappa coefficient of approximately 1. However, the remainings scarcely showed the significant correlation (Figure 1C).

### 3.3 Analysis of virulence factors

**FIGURE 2:**
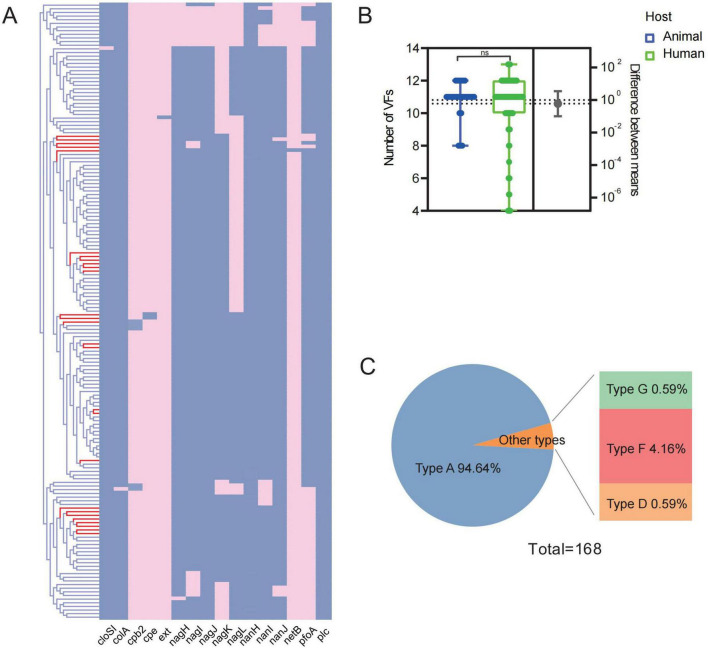
Results of virulence gene analysis. **(A)** Heatmap: prediction results of virulence genes; Dendrogram: clustering rows with similar data (red branches indicating strains of animal origin); **(B)** comparison of the number in virulence genes of *C. perfringens* from human and animal sources; **(C)** typing ratio of 168 *C. perfringens* strains.

To provide a comprehensive overview of the virulence factors of *C. perfringens*, we first profiled the virulence genes of isolates in the current study as well as the data from the database (Figure 2A). Among the 1all analyzed strains, type A *C. perfringens* presented as the majority of the *C. perfringens* (94.6%, 159/168). A total of 16 genes with significant nucleotide similarity to the known virulence factors among all analyzed strains. Specifically, all strains were identified the genes encoding *α-toxin* gene (*plc*), and over 70% of them carried the genes responsible for productions of toxins like *α-clostripain* (*cloSI*), *k-toxin* (*colA*), θ*-toxin* (*pfo*), hyaluronidase (*nagH*, *nagI*, *nagJ*, *nagK*, *nagL*), and sialidase (*nanH*, *nanI*, and *nanJ*). It was noteworthy that the 5 strains from human origin were found with abundant virulence factors. However, there was no significant difference (*P* > 0.05) in the numbers of virulence factors among the *C. perfringens* from either animal or human origin (Figure 2B). Virulence genes related to typing genes other than *plc* were detected in 9 strains, including one *C. perfringens* type D strain, and one *C. perfringens* type G strain along with seven strains of *C. perfringens* type F (Figure 2C).

### 3.4 Prophage prediction analysis

**FIGURE 3:**
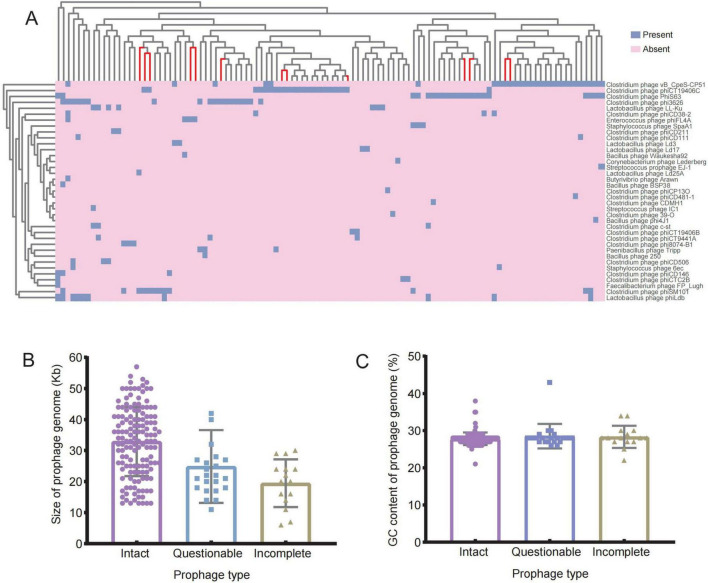
Prophages in the *C. perfringens* genome by the prophages. **(A)** Heatmap: the prophage profile of 168 *C. perfringens* genomes; Dendrogram: clustering rows with similar data (red branches indicate strains of animal origin). **(B)** Genome sizes of complete, putative and incomplete prophages; **(C)** GC content of intact, questionable and incomplete prophages.

A further sequence mining on the genome data of 168 *C. perfringens* was performed and indicated the presence of prophages in 108 of them. This collection includes 142 complete prophages, 23 putative prophages and 15 incomplete prophages. The 180 prophages were categorized into 37 distinct phage types, with the clostridial phage *vB CpeS-CP51* as the most prevalent type (29/180, 26.9%). This was the followed by the clostridial phage *phiCT19406C* (22/108, 20.4%), clostridial phage *PhiS63* (21/108, 19.4%) and clostridial phage *phi3626* (20/108, 18.5%) (Figure 3A). Further analysis of the prophage sequences (Figures 3B, C) showed that the *C. perfringens* prophage genome lengths were ranging from 6.7 to 66.4 kb and the GC contents were 21.7%–38.5%, respectively. The lengths of the complete prophage were around 13.4–57.6 Kb, and the GC contents were 21.7%–38.5%. The lengths of the putative prophage were 14–66.4 Kb and the GC contents were 26.9%–43.3%. Incomplete prophages were found with the smallest size of 6.7–30.8 Kb, whereas the GC contents were 22.5%–34.9%. Our statistical analysis, conducted using one-way analysis of variance (ANOVA) and Independent samples *t*-test in SPSS software, revealed that the numbers of prophages in human-derived strains were significantly higher than those in animal-derived strains (*P* < 0.01), but there were no significant differences in the lengths and GC contents (*P* > 0.05). The sequences of complete prophages were significantly longer than that of the suspected prophages and incomplete prophages (*P* < 0.05), while no significance was observed in terms of the GC contents (*P* > 0.05).

### 3.5 Molecular characteristics of *C. perfringens* isolates

**FIGURE 4:**
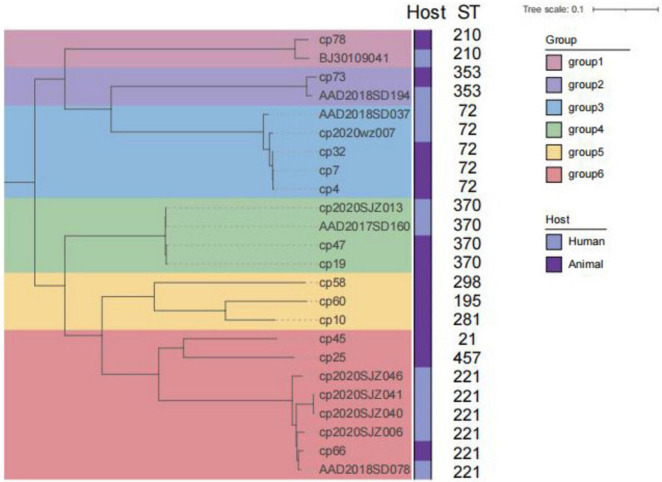
Phylogenetic tree based on the core genome of 24 strains of *C. perfringens*. The core genome of *C. perfringens* ATCC 13124 was used as outgroup. The six colors on the tree represent the six groups of analytical identification. The bootstrap values for each branch are all 1 (100%). The scale bar corresponds to 0.1 substitutions per site.

The genome sequences of *C. perfringens* isolates were subjected to the comparative genomics using PubMLST *C. perfringens* database, and 13 of 27 *C. perfringens* isolates were assigned to be the known ST. Nonetheless, 14 unknown STs have been identified in the *C. perfringens* isolates from the current study (Supplementary Table 6). In a more comprehensive view, there were a total of 103 STs among the *C. perfringens* strains including both our isolates and the isolates from the database. There were differences amongst STs between human- or animal-derived *C. perfringens* isolates. Among human-derived *C. perfringens* isolates, ST221 (*n* = 5) was the most dominant ST, followed by ST62 (*n* = 4) and ST408 (*n* = 4). the most dominant ST among food-animal-derived strains was ST72 (*n* = 3). However, strains of the same ST from different sources showed no significant clustering differences (Figure 4). The 10 major STs of animal-derived strains were classified into 6 clusters. ST221, ST72, ST370, ST210, and ST353 were also detected in human-originated strains.

### 3.6 Pan-genome analysis results

**FIGURE 5:**
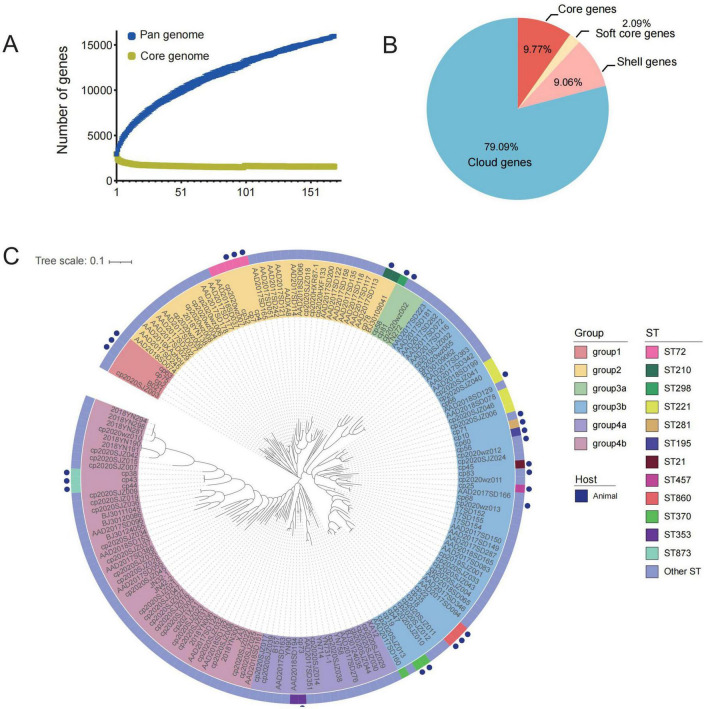
Results of pan-genomic analysis of *C. perfringens*. **(A)** Trend map of pan-genome; **(B)** the pan-genomes of these isolates were determined by comparing the pan-genomes, core genes, shell genes and cloud genes of the isolated *C. perfringens*; **(C)** phylogenetic tree analysis of the core genome of 168 strains of *C. perfringens*.

The genomic data of *C. perfringens* strains from this study and the database were analyzed and an open pan-genomes was determined with the pan-gene counts increasing proportionally (Figure 5A). The pan-genome of *C. perfringens* strains comprised 19,289 genes, encompassing 1,558 (9.8%) genes in the core genome, 333 (2.1%) genes in the soft core genome, 1,445 (9.1%) genes in the shell genome, and 15,953 (79.1%) genes in the cloud genome (Figure 5B). Genome-based phylogenetic analysis of the core genomes from the tested *C. perfringens* isolates revealed four major clusters, with the third and fourth clusters further classified into two subclusters (Figure 5C). Food-animal-derived source isolates were distributed within 6 clusters, indicating high diversity. However, there were also some interesting findings. For example, animal-derived isolates cp2, cp63 and cp75 together with human-derived ST421 strains (B021 and cp2020SJZ003) constituted a main lineage. Type A isolates (cp78 and BJ30109041, ST210) were closely related to the type F strain AAD2017SD113, which belongs to ST448 and has been associated with antibiotic-associated diarrhea in humans. It was worth noting that animal-derived *C. perfringens* cp47 and human-derived AAD2017SD160 showed high identity in Single Nucleotide Polymorphism (SNP). The SNP counts between animal-derived strains cp4, cp7 and cp66 and human-derived strains AAD2018SD078 as well as the cp2020wz007 were less than 395. This finding suggested that food animal served as a potential source of *C. perfringens* infection in humans. In addition, it was interesting that all animal-derived *C. perfringens* isolates in this study demonstrated a sharply different genomic traits with differences of more than 11,000 SNPs. This result indicated the potential diversity among *C. perfringens* genomes within the same region.

## 4 Discussion

Food poisoning caused by *C. perfringens* is frequently associated with contaminated meat products (Hu et al., 2018). In recent years, this pathogen has received increasing attention globally due to its potential threat to both human and animal (Talukdar et al., 2024). With the rapid development of whole-genome sequencing technology, the use of bioinformatics to study the pathogenicity, drug resistance, and genetic diversity of *C. perfringens* provides insights into the prevention and treatment of *C. perfringens* (Abdel-Glil et al., 2021a). In this study, we successfully isolated 27 strains of *C. perfringens* from the markets in Shandong, of which 26 strains were classified as type A and 1 strain as type G. The genome sizes ranged from 3.1 to 3.5 M bp, with GC contents ranging from 28 to 28.5%. This data, combined with the sequencing data of human-derived *C. perfringens* in China from database, was further employed for phylogenetic and functional analysis.

Numerous studies have revealed that the emergence of MDR *C. perfringens* poses a serious threat to public health (Khandia et al., 2021; Priya et al., 2023). Our study demonstrated the high levels of antibiotic resistance among *C. perfringens* isolates to tetracycline (100%, 27/27), lincomycin (59.3%, 16/27) and bacitracin (77.8%, 21/27). These high rates were likely owing to the tremendous usage of such antibiotics in poultry farming (Żbikowska et al., 2020). Among the 27 isolates, 24 (88.9%) exhibited resistance to three or more antibiotics. Previous studies have outlined that many *C. perfringens* isolates demonstrated resistance to a broad range of antibiotic classes including tetracyclines (2019), lincosamides (Udhayavel et al., 2017), aminoglycosides, and macrolides (Camargo et al., 2022). The global emergence and spread of MDR pathogens pose an unneglected challenge to therapeutic regimen based on antibiotics (Krishnamurthy et al., 2016). Previous investigations demonstrated that tetracycline resistance has been a prevalent drug-resistant phenotype among *C. perfringens* isolates, and most resistance genes are capable to transfer via plasmid conjugation (Park et al., 2010). Additionally, the resistance of *C. perfringens* to macrolide antibiotics has been well-documented (Baquero and Reig, 1992). The primary mechanism underlying this resistance is often mediated by the *erm* and *mef* genes. In our study, 19 (70.4%) of the 27 were found to carry the *erm* gene. Notably, the *erm* gene not only confers the resistance to macrolide antibiotics but also mediates the tolerance to lincosamide and streptogramin B. Furthermore, the dissemination of *erm* genes between species by mobile genetic elements has also been reported (Soge et al., 2009). Collectively, these findings suggested that tetracycline and macrolide resistance genes are prevalent in *C. perfringens* from both animal and human origins.

In this study, the discrepancy between phenotypes and genotypes of antibiotic resistances were observed except for tetracyclines and lincosamide drugs. This may be attributed to the presence of multiple resistance mechanisms. The relationship between ARGs and phenotypic resistance is complex and frequently oversimplified in public discourse. Although ARGs are routinely identified genomic sequencing technologies, their presence does not necessarily equate to phenotypic resistance. In fact, many ARGs require specific regulatory conditions to manifest resistance, such as when cloned into expression vectors or mutated to enhance expression (Perry et al., 2022). Moreover, environmental factors and genetic context also play crucial roles in determining whether ARGs confer phenotypic resistance. For example, the mobilization of ARGs by mobile genetic elements can lead to overexpression and resistance, while their presence in chromosomal locations may not result in significant phenotypic changes (Dai et al., 2022). We have learned that the combination of multiple software tools can provide a more comprehensive detection of genes resistance. For example, integrating the results from CARD and AMRFinderPlus (Feldgarden et al., 2021) can enhance the detection of resistance genetic determinants. We plan to incorporate AMRFinderPlus into our future analyses to ensure a more comprehensive detection of resistance genetic elements. This will provide additional support for our further analysis of the relationship between resistance genes and resistance phenotypes.

*C.perfringens* induces disease through the production of various toxins and enzymes. Notably, they are known to release over 20 distinct toxins, which serve as the primary mechanism for eliciting histotoxic pathogenesis in both humans and animals (Mehdizadeh Gohari et al., 2021). Most genes for encoding toxins in *C. perfringens*, particularly those relevant to strain typing, are conserved, thereby facilitating identification via PCR and sequencing (Feng et al., 2020). This study corresponded to this point. Type A and Type C of *C. perfringens* represent a substantial neglected threat to food hygiene and safety, as evidenced by the elucidation from this study. It is widely acknowledged that *cpa* is the primary toxin responsible for gas gangrene (Stevens et al., 1997), the *pfoA* gene plays a synergistic role alongside *cpa* in myonecrosis by promoting tissue destruction and stable persistence (Katayama et al., 2015). The toxin production is the significant traits for *C. perfringens* pathogenicity in human cases. Studies have shown that *pfoA*-positive strains are a common cause for the necrotic enteritis in infants (Kiu et al., 2023). Studies have shown that *C. perfringens* strains carrying *cpb2* are often found in the intestinal tracts of livestock and human (van Asten et al., 2010). Among human-derived strains, three strains of them were identified belonging to types D and F, respectively. According to the previous reports, the type D isolates mainly infect goats and sheep (Soge et al., 2009). These findings indicate that humans may be infected through contact with contaminated animals or food.

Prophages are recognized as significant contributors to the evolution of bacterial hosts (Fortier and Sekulovic, 2013). Through horizontal gene transfer, prophages are able to induce functional alterations in host bacteria by modifying the global transcriptomics. In our study, 108 of tested *C. perfringens* strains (63.9%) were found to carry complete prophages. Previous reports indicated that prophages constitute a relatively substantial portion of bacterial genomes, comprising up to 10%–20% of the genetic material of host bacteria (Casjens, 2003), In contrast, the prophages identified in this study only constitute approximately 1.04% of the entire bacterial genome, which is notably smaller than those observed in *E. coli* and *Salmonella* (Bobay et al., 2013). Our findings reveal that the most prevalent prophages within the *C. perfringens* genome are Clostridium phages that have been previously isolated and documented, aligning with the prior studies (Feng et al., 2020). Notably, the sequences of Clostridium phage *vB CpeS-CP51*, a prophage induced by mitomycin C (Gervasi et al., 2013), is the most abundant in the *C. perfringens* genome. This also suggested that tackling the spread of *C. perfringens* by inducing or lytic actions of prophages through mitomycin C is possible.

In this study, we found that the numbers of prophages in the human-derived strains were significantly higher than those in the animal-derived strains. This may be related to the unique selective pressures of the host environment, as the human body provides a complex and diverse habitat that favors the survival of bacterial strains with enhanced adaptability. Prophages, as mobile genetic elements, can provide bacteria with new genes that aid in survival, such as ARGs and VFGs. For instance, studies have shown that prophages in human-impacted environments, including those from human isolates, often carry a higher load of ARGs and VFGs, which can enhance bacterial fitness and virulence (Liao et al., 2024). Moreover, the high density and diversity of bacterial populations in human-associated habitats promote horizontal gene transfer (HGT). Prophages are known to facilitate HGT through transduction, allowing genes to be exchanged between different bacterial species. This process is particularly prevalent in human-derived isolates due to the close proximity and frequent interactions among diverse bacterial taxa within the human microbiome (Ku et al., 2025).

The identification of 10 novel STs in *C. perfringens* isolates holds significant epidemiological importance, especially in understanding cross-species transmission dynamics and zoonotic spillover risks. These STs may represent genetic adaptations that enhance pathogen fitness in diverse hosts or environments, potentially altering transmission efficiency between animals and humans (Fu et al., 2020). The emergence of novel STs may also reflect ecological niche expansion, similar to what has been observed in *New Campylobacter* linked to environmental persistence and low infectious doses (Cookson et al., 2024). From a public health perspective, the identification of these new STs underscores the need for revised diagnostic frameworks capable of detecting these emerging variants and for tailored interventions targeting high-risk interfaces, such as livestock markets or healthcare settings. Future studies should prioritize the functional validation of ST-specific virulence traits and evaluate their impact on outbreak trajectories using agent-based transmission models.

Our pan-genome analysis indicates that *C. perfringens* possesses an open pan-genome characterized by a relatively small core genome, suggesting high genomic plasticity. This finding is consistent with a recent study analyzing 372 *C. perfringens* genomes from diverse sources, which identified only 959 core genome genes within a pan-genome of 35,876 genes, accounting for just 2.7% (AlJindan et al., 2023). Similarly, another study demonstrated that *C. perfringens* strains carrying the *becAB* genes were distributed across distinct lineages and horizontally transmitted via a conserved Pcp plasmid (Fang et al., 2025), further confirming the species’ genomic plasticity. To address potential annotation errors encountered during our use of Roary, we implemented manual correction of high-frequency false-positive gene clusters. These quality control steps helped reduce inaccuracies in core genome representation. Furthermore, we observed that Panaroo offers significant advantages in resolving annotation errors during pan-genome analysis. Studies indicate that compared to Roary, Panaroo achieves more accurate identification of pairwise SNPs and effectively corrects diverse types of annotation errors through its graph-based algorithm (Tonkin-Hill et al., 2020). We have incorporated Panaroo into our future research plans, particularly for scenarios involving complex genome rearrangements or large-scale pan-genome studies.

This implied that *C. perfringens* genomes are highly plastic, plausibly acquiring favorable genetic materials from the surrounding environment for enhancing its adaptability (Feng et al., 2020). Notably, the utilization of pan-genome analysis has proven effective in elucidating the evolutionary trajectory of pathogenic bacteria (Stott and Bobay, 2020). Based on the core genome analysis of analyzed *C. perfringens* strains, we found that shared STs exist between animal-derived and human-derived strains, specifically ST221, ST72, and ST370. Of note, ST72 was isolated from a farm in Tai’an, Shandong province, as reported in previous studies (Xiu et al., 2020). Studies have shown that human-derived isolates are closely related to chicken-derived strains isolated from retail markets (Xu et al., 2021). It is important to highlight that the SNP sites of strains from different origins within the shared ST types only exhibit minor differences. It sheds the light on the possibility for outbreaks caused by *C. perfringens* (Abdel-Glil et al., 2021b).

This study provides valuable insights into the genetic and epidemiological characteristics of *C. perfringens* in China, particularly in Shandong Province, the country’s largest poultry-producing region. However, its geographically restricted scope limits the ability to comprehensively assess the pathogen’s global transmission and evolutionary dynamics. Specifically, the global distribution and genetic diversity of *C. perfringens* are known to vary across regions due to differences in environmental conditions, host interactions, and agricultural practices (Ba et al., 2024). Consequently, reliance on Chinese strains alone may not fully capture the evolutionary trajectories or transmission patterns observed in other parts of the world. For example, strains adapted to China’s intensive farming systems may exhibit distinct genetic or phenotypic traits compared to those from regions with alternative livestock management strategies (Yanxia et al., 2025). Therefore, while this work advances our understanding of *C. perfringens* within a Chinese context, its relevance to broader global patterns of zoonotic spread and pathogen evolution remains constrained. To fully understand the pathogen’s global adaptability and transmission mechanisms, future studies will include strains from more diverse regions. This approach will improve the generalizability of our findings.

In this study, we conducted a comprehensive whole-genome analysis of 27 *C. perfringens* isolates from the markets in Shandong, China. They were subjected to a comparative genomic analysis with data of 141 *C. perfringens* database. By profiling the molecular characteristics, the understanding toward the genetic diversity of antibiotic resistances and virulences among the *C. perfringens*. Eventually, the in-depth analysis also shed the light on the evolutionary trajectory and transmission dynamics of *C. perfringens*, as well as the emerging risks of these pathogens to livestock industry and public health.

## Data Availability

The datasets presented in this study can be found in online repositories. The names of the repository/repositories and accession number(s) can be found in this article/Supplementary material.

## Footnotes

1
https://github.com/tseemann/shovill

2
https://clsi.org

3
http://www.mgc.ac.cn/cgi-bin/VFs/v5/main.cgi

4
https://card.mcmaster.ca/

5
https://phastest.ca/

6
https://pubmlst.org/organisms/clostridium-perfringens

7
https://cge.food.dtu.dk/services/CSIPhylogeny/

8
https://itol.embl.de/
